# Is the Observed Decrease in Body Temperature During Industrialization Due to Thyroid Hormone-Dependent Thermoregulation Disruption?

**DOI:** 10.3389/fendo.2020.00470

**Published:** 2020-07-22

**Authors:** Pieter Vancamp, Barbara A. Demeneix

**Affiliations:** UMR 7221 Molecular Physiology and Adaptation, Centre National de le Recherche Scientifique—Muséum National d'Histoire Naturelle, Cedex 05, Paris, France

**Keywords:** body temperature, thyroid hormone, thyroid hormone metabolism, thermoregulation, endocrine disrupting chemicals, industrialization, homeostasis

Protsiv et al. used three sets of data to demonstrate that human core body temperature had decreased by 0.03°C per decade since the industrial revolution in the US (1). They proposed that a 1.6% temperature drop over a period of almost 200 birth years had occurred. Anthropometrics, gender, or race were excluded as potential factors. The authors postulated that the principle contributor to this reduction was reduced inflammation, reflecting better, healthier environments and improved hygiene measures (1). Although hygiene has increased and hence reduced death from infectious disease, other factors in our environment have also changed significantly. Here we propose another plausible and potentially testable mechanism, that of the contribution of factors interfering with thyroid hormone (TH) metabolism.

TH is an essential physiological cue that acts at central and peripheral levels to affect internal temperature in endotherms (2). Humans strive to live at thermoneutral conditions, in which peripheral muscle metabolism generates sufficient heat as a by-product to maintain temperature without the need for additional heat-generating mechanisms (3). For us, the resting metabolic rate (RMR) is thus a crude proxy for core body temperature. TH directly affects the RMR by altering mitochondrial biogenesis and oxidative phosphorylation via TRα1, the principle TH receptor isoform in muscle (Figure 1) (4). TH fluctuations within the normal range alter the RMR in humans (5, 6), suggesting that subtle changes in TH homeostasis could have consequences for body temperature. Recent data indicate that TH also safeguards core body temperature at the central level. Peripheral sensors relay temperature information to hypothalamic nuclei where temperature-sensitive neurons further calibrate body temperature to a pre-fixed set point (7, 8). Mice in a thermoneutral environment that were given high doses of TH either systemically, or via direct hypothalamic injection, had an acutely elevated central temperature set point (9–11). These central TH effects are most-likely an example of pyrexia, or a controlled set point change, as opposed to uncontrolled hyperthermia (9). In mice lacking TRα1, the central thermostat was downregulated (12, 13) in addition to reduced metabolism and impaired heat dissipation (14). Of note, in small mammals like mice, TH also activates brown adipose tissue to generate additional heat through adaptive thermogenesis (15), a process that is possibly less relevant in healthy adult humans, though this point is under continual debate (16).

**Figure 1:**
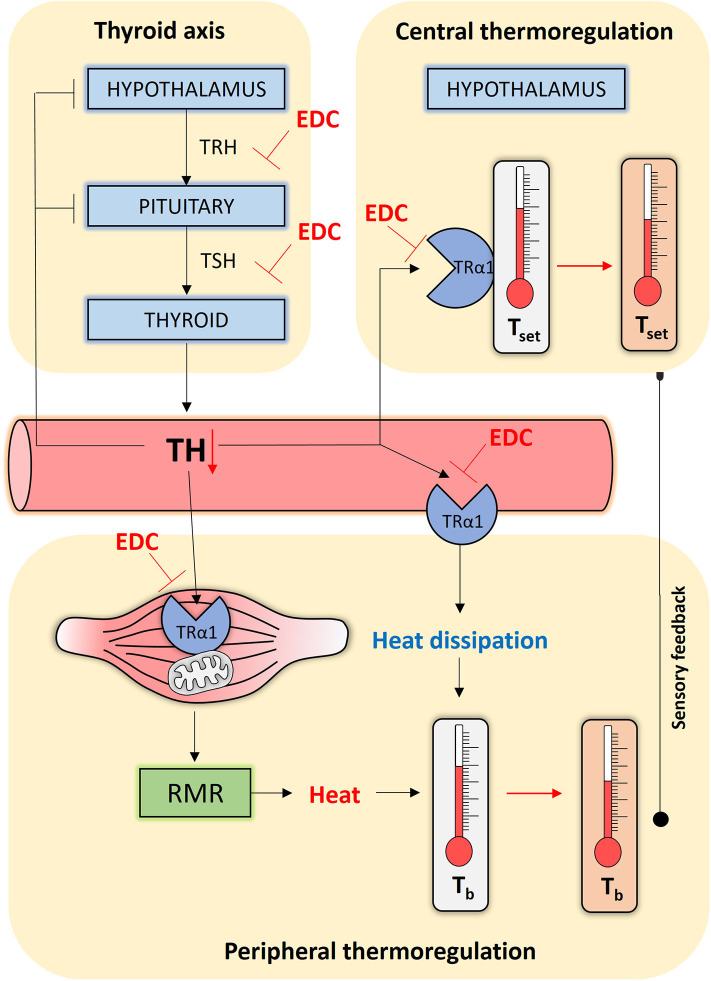
Simplified scheme of how endocrine disrupting chemicals (EDCs) might interfere with human thermoregulation. The Hypothalamic-Pituitary-Thyroid (HPT) axis, or thyroid axis, consists of neurons synthesizing thyrotropin-releasing hormone (TRH) that stimulates thyrotropes in the pituitary to release thyrotropin (TSH). TSH stimulates the thyroid gland to produce and secrete the thyroid hormones (THs), which negatively feedback at the level of the HPT axis. THs reach central and peripheral organs via the circulation. At the level of the hypothalamus, TH programs and safeguards the temperature set point (T_set_). At the level of the muscle tissue, TH alters the resting metabolic rate (RMR) by acting on oxidative phosphorylation and mitochondrial biogenesis, generating heat in the process that maintains core body temperature (T_b_). TH may additionally affect heat dissipation by regulating vasoconstriction. All the actions listed above, other than the thyroid axis set point, occur primarily through TRα1. EDCs can interfere at every point in this interconnected network and disrupt TH homeostasis or interfere with the activation of TRα1, causing impaired thermoregulation control through persistent temperature set point changes, a reduced RMR and altered heat dissipation.

But how do these actions affect core body temperature in humans? Back in 1894, a medical doctor T.C. Railton observed the significant impact TH can have on body temperature (17). His patient was a 14-year-old hypothyroid boy with an abnormally low body temperature of 35°C. After giving 36 grains of sheep's thyroid gland (thyroxin or T_4_ was not isolated until 1914 by Edward Kendall), his temperature rose to a staggering 39.1°C. Adjusting the dose to 5 grains twice a day normalized his body temperature to approximately 37°C (17). This one-person trial proved that the amount of thyroid tissue, and thus THs, dose-dependently alters body temperature. Similarly, the doctor treating the first-ever myxedema patient by hypodermic injection of thyroid extract, noted that her body temperature increased by several degrees (18). More recently, intravenous injection of high doses of levothyroxine reversed the low body temperatures (34–35.8°C) of seven comatose myxedema patients to more than 36°C (19).

Another intriguing case that demonstrates these temperature effects of TH is *Wilson's Temperature Syndrome*, first described in 1990. Patients display a form of chronic fatigue syndrome with symptoms that are hallmarks of hypothyroidism, as well as a lower-than-normal body temperature. Giving cyclic oral supra-physiological doses of T_3_ (up to 30 μg/day) elevated the average body temperature from 36.34 to 37°C in 11 tested patients (20). While the temperature fluctuations in Railton's case were in the range of several degrees, Friedman's data show that smaller doses of THs can induce body temperature changes around 0.5°C. It would be very interesting to know whether subclinical hypothyroidism, a condition marked by lower but within the normal range levels of TH that affects around 10% of the population (21), also alters average body temperature in an even narrower regimen. So far, only one cross-sectional study has been reported on the subject (22). This study included 306 subclinically hypothyroid subjects, however, the authors observed no body temperature differences with euthyroid individuals. Three potential caveats need to be mentioned here, first that temperature measurements only included one digit after the decimal point, whereas in the study carried out by Protsiv et al. (1) the authors extended their findings to two digits. The second is the small number of subclinical hypothyroid patients in the cohort tested and third, to be classed as subclinically hypothyroid, T_4_ levels must lie within the normal range, whereas TSH levels are elevated. As the cut-off range for elevated TSH varies according to region, but can easily be a 10 fold span, this emphasizes the difficulty of classing someone as subclinically hypothyroid.

If a similarly deregulated TH homeostasis lies at the base of the gradual temperature decrease during industrialization, what could have disrupted TH metabolism in the first place? A number of factors have changed in our environment since the industrial revolution. Chemical production has increased 300-fold since 1970, both in terms of diversity and quantity (UNEP, 2013—*Global Chemicals Outlook - Toward Sound Management of Chemicals*). These so-called endocrine disrupting chemicals (EDCs) affect each and every one of us in a gender-, race-, and anthropometric-independent manner (23). Apart from the range of effects on our daily body physiology, especially that of future generations, we hypothesize that the long-term use of chemical compounds might even have changed our own body's homeostatic mechanisms, including the RMR.

A myriad of studies have now unequivocally proven that this constant exposure to low doses of chemical mixtures can deregulate the thyroid axis, and alter human body homeostasis (24). The most evident adverse effect comprises impaired neurodevelopment, and human epidemiological data provide two lines of worrying evidence in that regard (25). First, deviating maternal TH levels, but within the normal range during fetal development lead to irreversible, structural changes in the central nervous system of infants and unfavorable outcomes such as lowered intelligence quotient (25, 26). Second, some of the investigated widely-used chemicals induce changes in TH levels (27) of the magnitude seen in the study of Korevaar et al. (26).

We speculate that chemically disrupted TH signaling interferes with the development and functioning of central thermoregulation (Figure 1). A population of parvalbuminergic neurons in the anterior hypothalamic area integrate temperature information to control heart rate and blood pressure (28). As abnormal maternal TH levels disrupt neuronal migration and differentiation, resulting in permanent hypertension and temperature-dependent tachycardia (29) it is possible that developmental disruption of maternal/fetal TH signaling by EDCs could adversely affect the fine-tuning of the temperature set point in a similar way. A clear-cut example is the persistent organochlorine pesticide dichlorodiphenyltrichloroethane (DDT) that is still found in significant levels in breast milk and amniotic fluid. Average concentrations of 3.49 ng DDT per g lipid, and 198.34 ng/g of its main metabolite DDE, were detected in pregnant women of a 2003–2004 cohort in the NANHES study (30). DDT is a very potent thyroid disruptor in animals (31, 32) and humans (33). Female mice exposed to 1.7 mg DDT per kg body weight during a two-week perinatal period, had a permanently lower core body temperature up until the age of 5 months (34). When combined with a high-fat diet, average body temperature in the adult mice dropped even more, causing many other complications such as insulin resistance and altered glucose/lipid metabolism (34). In obese people too, body temperature is lower than average (35). These comorbidities are associated with metabolic syndrome, a global health hazard that is mainly considered a non-communicable disease primarily caused by a sedentary lifestyle and calorie-rich diet, but also endocrine disruption. Such data suggest developmental misprogramming due to chemical exposure could be another trigger predisposing to metabolic syndrome (36).

Another possibility is that EDCs affect adult thermoregulation by interfering with TH-dependent central and peripheral thermoregulation in the adult (Figure 1) (37). Key to our reasoning is the fact that we are constantly exposed to low doses of complex EDC mixtures (38). On the one hand, EDCs can indirectly affect thermoregulation by disrupting thyroid axis-regulated TH homeostasis. Polychlorinated biphenyls, polybrominated diphenyl ethers, perchlorate, bisphenols, phthalates, pesticides, and perfluoroalkyls all cause persistent changes in circulating TH levels in animals and humans (39, 40). For example, the individuals of a cohort of 679 male pesticide applicators that were continuously exposed to the highest doses of the insecticide aldrin or the herbicide pendimethalin had higher TSH and lower T_4_ levels (41). These factors could thus alter the RMR or alter hypothalamic TH signaling (37). On the other hand, EDCs can interfere with the availability of THs to activate TRs (Figure 1). Adult mice shortly exposed to 100 μg/kg bisphenol A, an antagonist of TH action (42), displayed impaired muscle metabolism and lower body temperatures (43). Chemicals can also deregulate central control of metabolism and the energy balance (37), but whether they do so by disrupting local TH signaling remains to be investigated. In addition, EDCs also affect other hormone axes, as for instance glucocorticoids that are intimately implicated in metabolism and heat generation (44).

Data linking (developmental) chemical exposure, disrupted TH signaling and permanently reduced core body temperature are scarce, and need additional testing. A well-planned experiment should aim to causally link this chain of events to provide better proof for this paradigm. We recommend exposing mice to a well-known TH disrupting chemical (or mixture of chemicals) during perinatal development and follow core body temperature during postnatal life up until the adult stage. Simultaneously exposing mice to T_3_ during specific intervals could reveal critical time windows for establishment of the hypothalamic temperature set-point. Data should be coupled to mapping the neuroanatomical and (epi)genetic landscape in the (developing) hypothalamic nuclei. In addition, adult-onset exposure of adult wild-type and TRα1-deficient mice might reveal at which levels a particular EDC is most liable to induce disruption of thermoregulation. Such actions could be at the peripheral level by interfering with the RMR in muscle tissue, through impairing heat dissipation, or at the central level by altering the temperature set point, or possibly a combination of the above. To mimic effects on human thermoregulation best, experiments should be performed at thermoneutral conditions (45). Testing for dose-dependent effects on temperature will be subtle and requires a high “n” number per group. In the meantime, collecting data from large cohorts of patients with thyroid conditions, for instance of children born to (sub)clinically hypothyroxinemic mothers, could unravel similar correlations with offspring body temperature. To strengthen our hypothesis further, we should ideally have data from pre-industrialization or from emerging countries that have not yet fully industrialized to uncover possible trends. However, to the authors' knowledge no such data is available.

Our arguments remain merely speculative, but suggest yet another plausible mode of action of how EDCs can interfere with whole body homeostasis. While we are beginning to understand the complex mechanisms by which industrial chemicals endanger human health and wildlife preservation, we can only surmise the consequences of long-term exposure. With the advent of global crises such as climate change or the recent COVID-19 pandemic, it is however imperative to fully grasp the dynamic interaction between these factors so as to protect future generations from these threats that include chemical pollution.

## Author Contributions

BD worked out the concept of the paper. PV and BD both wrote the paper. All authors contributed to the article and approved the submitted version.

## Conflict of Interest

The authors declare that the research was conducted in the absence of any commercial or financial relationships that could be construed as a potential conflict of interest.
